# Chitinases and Imaginal disc growth factors organize the extracellular matrix formation at barrier tissues in insects

**DOI:** 10.1038/srep18340

**Published:** 2016-02-03

**Authors:** Yanina-Yasmin Pesch, Dietmar Riedel, Kapil R Patil, Gerrit Loch, Matthias Behr

**Affiliations:** 1 Institute for Biology and Translational Center for Regenerative Medicine (TRM), University of Leipzig, 04103 Leipzig, Germany; 2 Life & Medical Sciences Institute (LIMES), University of Bonn, 53115 Bonn, Germany; 3 Max Planck Institute for Biophysical Chemistry, Electron microscopy group, 37077 Göttingen, Germany

## Abstract

The cuticle forms an apical extracellular-matrix (ECM) that covers exposed organs, such as epidermis, trachea and gut, for organizing morphogenesis and protection of insects. Recently, we reported that cuticle proteins and chitin are involved in ECM formation. However, molecular mechanisms that control assembly, maturation and replacement of the ECM and its components are not well known. Here we investigated the poorly described glyco-18-domain hydrolase family in *Drosophila* and identified the *Chitinases (Chts)* and *imaginal-disc-growth-factors (Idgfs)* that are essential for larval and adult molting. We demonstrate that *Cht* and *idgf* depletion results in deformed cuticles, larval and adult molting defects, and insufficient protection against wounding and bacterial infection, which altogether leads to early lethality. We show that *Cht2/Cht5/Cht7/Cht9/Cht12* and idgf1/idgf3/idgf4/idgf5/idgf6 are needed for organizing proteins and chitin-matrix at the apical cell surface. Our data indicate that normal ECM formation requires *Chts*, which potentially hydrolyze chitin-polymers. We further suggest that the non-enzymatic *idgfs* act as structural proteins to maintain the ECM scaffold against chitinolytic degradation. Conservation of Chts and Idgfs proposes analogous roles in ECM dynamics across the insect taxa, indicating that Chts/Idgfs are new targets for species specific pest control.

The epithelial apical extracellular matrix (ECM) controls development and structural maintenance of organisms, organ morphogenesis, wound healing, cell signaling, and the local defense barrier against a hostile environment^1^,^2^. Numerous events, such as tissue metamorphosis and remodeling, require dynamic alterations and degradations of the ECM architecture. Epithelial cells secrete essential components, such as proteins, lipids and polysaccharides, into the extracellular space where they form a complex network of proteins and proteoglycans. These extracellular components, together with apical membrane bound proteins, organize the ECM as a first barrier at the epithelial cell surface^2^,^3^,^4^,^5^.

In mice and humans mutations of the extracellular collagen and the cornified envelope have been linked to skin disorder diseases, characterized by skin fragility, painful wounding and accompanied skin infections. The skin layers establish stability and an essential protective barrier by crosslinking of extracellular components^5^,^6^. In chitinous invertebrates, the ECM barrier is represented by the cuticle, an exoskeleton that covers the body and internal organs as an epithelial surface layer^7^,^8^. The cuticle is a highly organized ECM structure composed of the polysaccharide chitin and a variety of proteins and enzymes^4^. This exoskeleton is essential for body size control, epithelial barrier formation, epidermal wound healing and protects cells from direct contact with pathogens, toxins or pesticides^1^,^9^,^10^. It further provides barriers for maintaining homeostasis of body fluids^11^.

Genes involved in the biosynthesis and secretion of chitin-ECM components have been studied intensively in *Drosophila*^4^,^12^,^13^. Biosynthesis is assisted by conserved factors to control precise spatial and temporal fine-tuning of chitin deposition in the organs^14^. The chitin-polymers arise at the apical cell surface, where they establish a lamellate and tight chitin-ECM^15^. Recently, we showed that the chitin-ECM formation is coordinated within the cuticle assembly zone, which is a prominent part of the cuticle at the apical cell surface^16^. Several proteins and enzymes are located within the cuticle assembly zone in order to establish a tightly packed ECM. This involves the Obstructor-A (Obst-A) protein^17^ that binds chitin-fibrils to form a scaffold for localizing chitin deacetylases Serpentine (Serp) and Vermiform (Verm), to mature physical and chemical properties of the chitin-matrix^16^,^18^,^19^. The protection of the newly synthesized cuticle is mediated by the Knickkopf (Knk) protein in *Tribolium* and a similar role in *Drosophila* has been discussed recently^20^,^21^.

Chitin-polymers may spontaneously assemble into microfibrils of various lengths and diameter^22^. At the apical cell surface they need to be organized into a tight and compact ECM architecture. This process of chitin-ECM assembly and replacement is repeated several times due to cuticle molting and upon wounding and thus may involve a strict genetic control. Genes that control the developmental dynamics of chitin-ECM replacement are poorly known. The glycosylhydrolases were shown to be active in hydrolyzing chitin-polymers of different sizes and have been found in many species, including plants, insects and mammals^23^,^24^,^25^,^26^,^27^,^28^,^29^. In arthropods glycosylhydrolases are encoded by an evolutionary conserved multigene family, referred to as *family18* due to the characteristic glyco18 domain. The *family18* comprises numerous Chitinases (Chts) and Imaginal-disc-growth-factors (Idgfs)^26^,^27^, initially identified from *Drosophila* imaginal disc cells^30^. The glyco18 domains of Chts are catalytically active and may assist in the hydrolysis of simple or complex chitin-polymers, while glyco18 domains of Idgfs are inactive^26^,^30^,^31^,^32^. However, the roles of many *family18* members in ECM organization and accompanied dynamics of the cuticle remained largely unknown.

We systematically investigated *family18* members in ECM formation in *Drosophila*. By individually knocking down genes in the cuticle secreting tissues, we show that a large number of the *Chts* and *idgfs* are involved in cuticle molting during larval as well as in pupal stages. This is supported by gene specific spatial-temporal expression profiles and by developmental lethality profiles upon gene knockdowns. Moreover, we demonstrate that many *Chts* and *idgfs* genes are required also during the intermolt stages for forming the epidermal cuticle in order to organize the outermost protective barrier, since knockdown mutants are highly susceptible to mechanical stresses and bacterial infections. Investigating the molecular network in *Cht2* and *idgf6* RNAi induced mutants, which showed strongest lethality and most severe cuticle defects, we propose that certain Chts are involved in organizing the chitin-matrix scaffold, which is essential for further enzyme-mediated ECM maturation and cuticle thickness. In contrast to Chts, the non-enzymatic Idgfs play an essential role in the protection of the newly synthesized cuticle matrix against degradation in order to stabilize and expand the ECM size in the larvae.

## Results

The *Drosophila family18* comprises sixteen members consisting of ten *Chitinase* (*Cht2, Cht4, Cht5, Cht6, Cht7, Cht8, Cht9, Cht11, Cht10, Cht12)* and six *idgf* genes (*idgf1, idgf2, idgf3, digf4, idgf5* and *idgf6)*^25^,^27^. Their protein domain arrangements and amino acid alignments are conserved across insects and have been studied elsewhere^26^,^27^,^33^. The Cht protein domains include N-terminal Signal-Peptides, a variable number of glyco-18 domains and protein specific transmembrane- and chitin-binding-domains. The Idgf proteins contain one N-terminal Signal-Peptide for protein secretion and one glyco-18 domain (Supplementary Fig. S1a). Previously, it was shown by an RNAi injection screen in *Tribolium castaneum* that *Cht5, Cht10* and *idgf4* genes are involved in the molting process^34^. However, the role of many other *Chts* and *idgfs* in cuticle molting remains elusive.

### A large number of distinct *Chts* and *idgfs* genes control larval and adult cuticle molting

**Figure 1:**
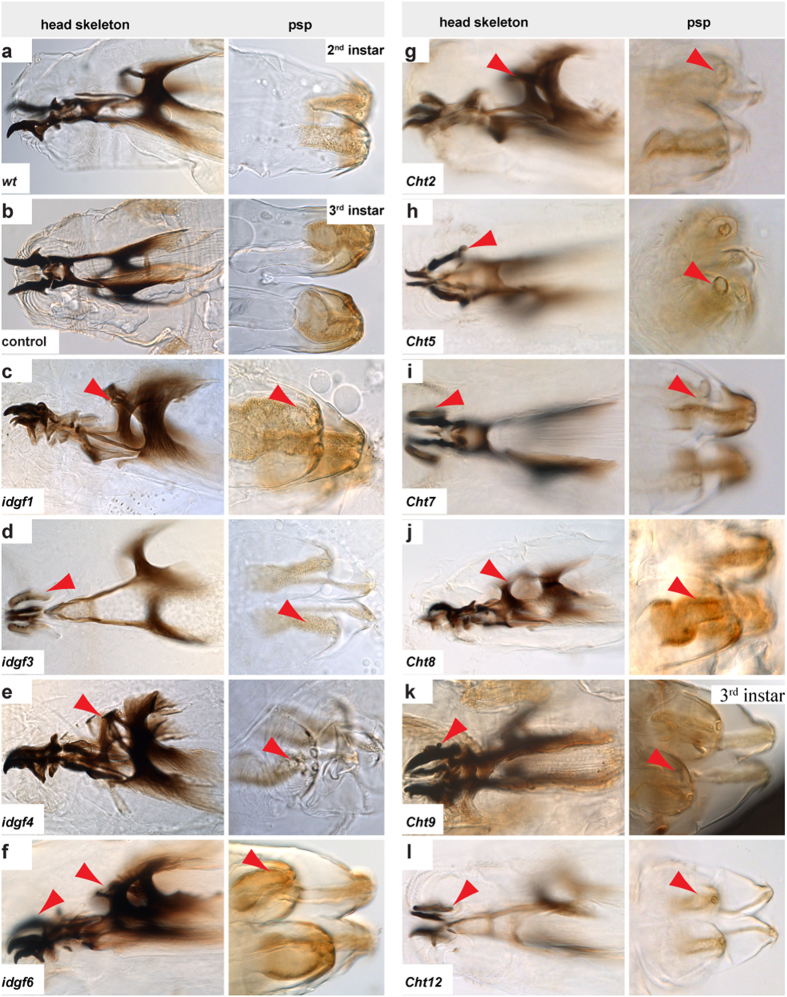
*Chts* and *idgfs* control larval cuticle molting. Larval molting defects visualized by bright field microscopy of cuticle preparations. (**a**,**b**) In second (*wt*) and third instar stages *(69B-Gal4* control animals) larvae contained single cuticles of mouth hooks, head skeleton (left panel) and posterior spiracles (psp) (right panel). (**c–f**) In contrast, individual *idgf1, idgf3, idgf4* and *idgf6* RNAi-mediated knockdown resulted in cuticle molting defects. Mutant larvae contained double cuticles (arrowheads), including old and new cuticles, of mouth hooks, head skeleton and posterior spiracles. (**g–l**) Similarly the individual *Cht2, Cht5, Cht7, Cht9, Cht8 and Cht9 and Cht12* knockdown led to double cuticles (arrowheads). It is of note that *Cht9* knockdown resulted in double cuticle only when larvae molt into third instar.

**Figure 2:**
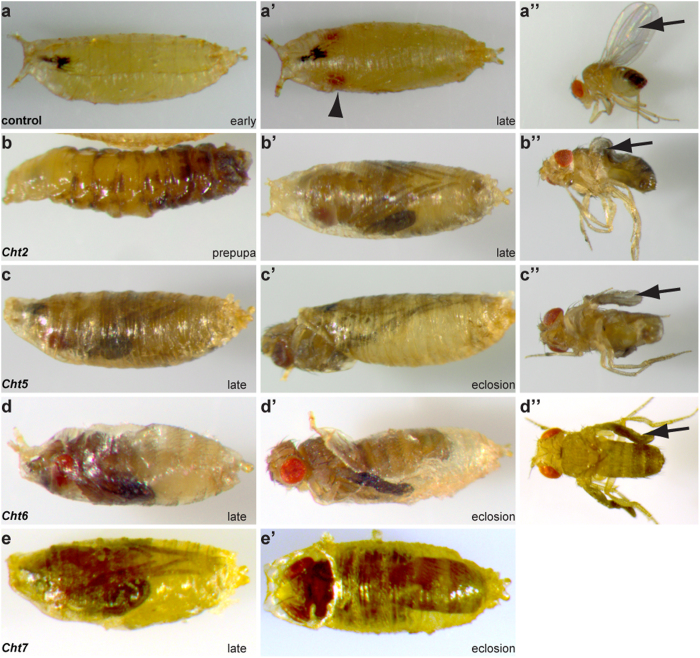
*Chts* coordinate pupal-adult molting and wing extension. Pupal arrest and wing extension defects were documented by bright field microscopy. (**a–a’**) Early (**a**) and late (**a’**) *69B-Gal4* control pupae. The late pupa contained characteristic red colored eyes (arrowhead). The newly hatched *69B-Gal4* control fly completed wing extension ((**a’**), arrow). (**b–b”**) The RNAi-mediated *Cht2* knockdown resulted in early (**b**) and late pupal (**b’**) lethality. Very few adult flies hatched but showed wing extension defects (**b”**) and died. (**c–e’**) The individual *Cht5, Cht6 and Cht7* knockdown caused late pupal lethality (**c**–**e**) and eclosion defects (**c’**–**e’**) in which adult flies are trapped inside the pupal cuticle. Hatched adult *Cht5* and *Cht6* knockdown flies revealed wing extension defects (**c”**,**d”**).

In order to analyze *Cht* and *idgf* genes systematically in cuticle forming tissues, we performed individual gene specific transgenic RNA interference (RNAi)-mediated knockdown in *Drosophila* using lines of the very efficient (see below) Vienna stock collection (www.vdrc.at)^35^. For all experiments described below UAS-*RNAi* was driven in cuticle-forming organs with the help of the *69B-Gal4* driver line (Supplementary Fig. S1b-d). *Wild-type (wt)* and *69B-Gal4* animals were taken as control. Our knockdown studies identified a large subset of genes required in larval cuticle molting and a distinct set essential in pupal-adult molting (Figs 1,2). The *Cht2,5,7,8,9,12* and the *idgf1,3,4,6* knockdown mutants showed characteristic double cuticles of head skeleton and posterior spiracles in second and third instar larvae although with variances in the severity of phenotypes (Fig. 1). Individual depletion of *Cht2,5,6,7* genes resulted in pupal molting defects and interfered with normal wing extension (Fig. 2). Pupal molting defects were not observed upon knockdown of *idgf* genes. Monitoring animals for twelve days, from egg laying until adulthood, demonstrated that *Cht* and *idgf* reduction caused larval and pupal lethality (Supplementary Fig. S1f,g). Relative gene expression profiles compared to first instar larvae showed up-regulation for *Cht8,9,12* at second and third larval stages and for *Cht2,5,6,7* at pupal stages (Supplementary Fig. S2) which partially reflects their time dependent knockdown phenotypes during molting (Figs 1,2). In contrast, *idgf* genes are expressed at rather constant levels throughout larval and pupal development (Supplementary Fig. S2). In summary, these studies show that numerous *Cht* and *idgf* genes are essential for cuticle molting throughout development. Importantly, among the *family18* members, *Cht2* and *idgf6* knockdowns resulted in the strongest lethality during larval stages (Supplementary Fig. S1f, g).

### *Chts* and *idgfs* are essential for organizing the exoskeletal barrier

**Figure 3:**
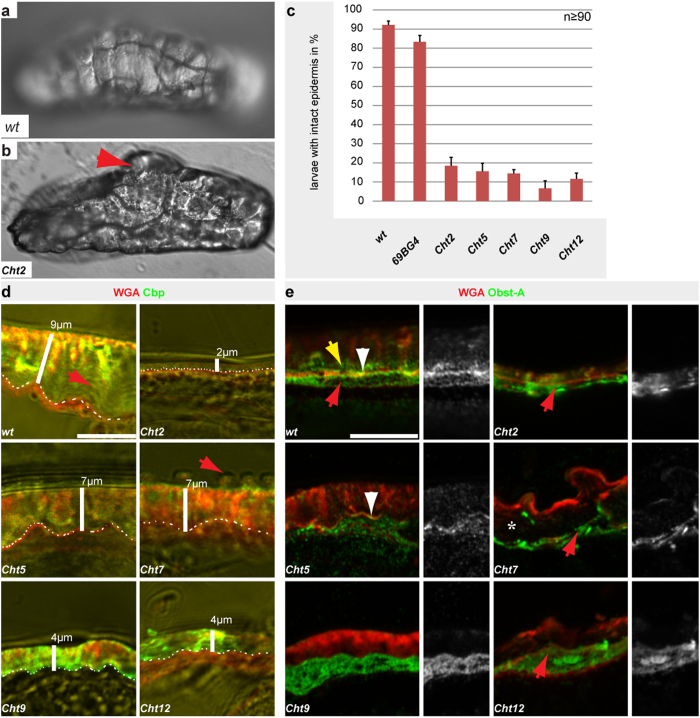
*Chts* organize the cuticle barrier and ECM formation at the apical surface. Phenotypic data for *Cht* RNAi lines combined with the tissue specific *69B-Gal4* driver line. (**a**,**b**) Epidermal cuticle barrier on first instar larvae after puncture wounding visualized by Nomarski-optic. *wt* larvae (**a**) survived gentle lateral puncture wounding while *Cht2* knockdown (**b**) resulted in epidermal burst, severe bleeding, organ spill out and immediate lethality (arrow). (**c**) The histogram indicates epidermal cuticle integrity defects in wounded *Cht2, Cht5, Cht7, Cht9, and Cht12* knockdown larvae; n >50 individuals; error bars indicate standard deviations. (**d**) Confocal and bright field microscopy of cross-sections of third instar larvae show the epidermal chitin-matrix by Cbp (green) and the apical surfaces by WGA (red). In *wt* larvae the chitin-matrix detected by Cbp was the most prominent part of the cuticle and appeared in a stratified organization (red arrow) resembling procuticle chitin-lamellae. *Cht2, Cht5, Cht7, Cht9* and *Cht12* knockdown led to a deformed stratified chitin-matrix. In *Cht2, Cht9* and *Cht12* knockdown larvae the chitin-matrix was narrowed as indicated by the white bars. It is of note that *Cht7* knockdown larvae showed unusual blisters (red arrow). For gene knockdown n ≥ 15 individuals were analyzed. White dashes indicate the apical cell surface. (**e**) Effects of *Cht* knockdown on the chitin-matrix organizer Obst-A in third instar larvae epidermis visualized by confocal microscopy. In *wt* larvae Obst-A was enriched at the apical cell surface (white arrowhead) marking the cuticle assembly zone^16^. Additional intracellular (red arrow) and extracellular (yellow arrow) Obst-A was detected. In contrast, *Cht2, Cht7, Cht9* and *Cht12* knockdown led to loss of Obst-A enrichment at the apical cell surface. Red arrow points to intracellular Obst-A expression. The extracellular Obst-A was either mislocalized at the entire chitin-matrix in *Cht2* mutants or strongly reduced (*Cht7, Cht9, Cht12*). In *Cht5* knockdown larvae Obst-A was only partially apically enriched (arrowhead). Note that the cuticle was detached from epidermal cells (asterisk) in *Cht7* knockdown. Scale bars represent 10 μm.

**Figure 4:**
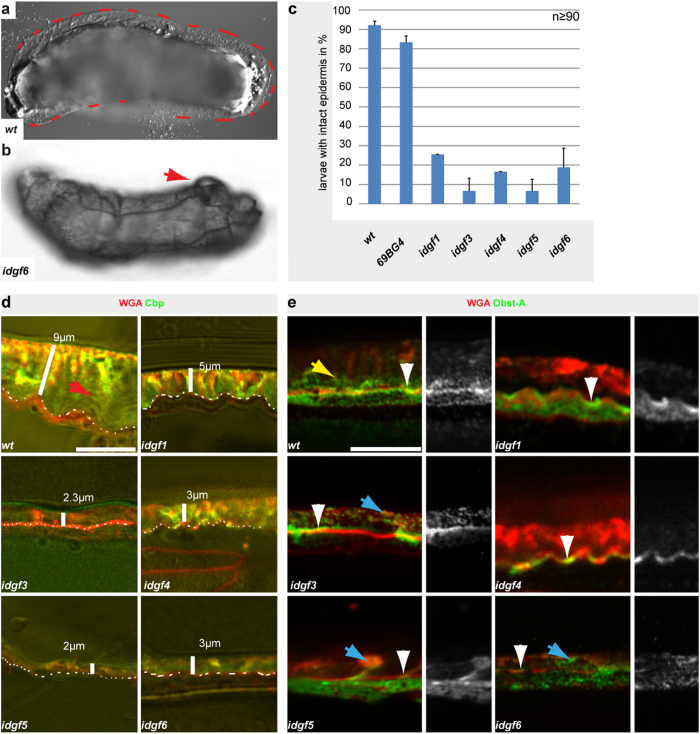
*Idgf*s are required for apical cuticle barrier and ECM formation. Phenotypic data for *idgf* RNAi lines combined with the *69B-Gal4* driver. (**a**,**b**) Cuticle barrier was analyzed in first instar larvae, as visualized by Nomarski-optic. *wt* larvae (**a**) survived gentle lateral puncture wounding while *idgf6* knockdown resulted in epidermal rupture (arrow), organ spill out and lethality. (**c**) The histogram indicates that the epidermal cuticle barrier integrity of wounded larvae was severely affected as a result of individual *idgf1, idgf3, idgf4, idgf5 and idgf6* knockdown; n > 50 individuals; error bars indicate standard deviations. (**d**) Confocal and bright-field microscopies of third instar larvae; the epidermal chitin-matrix was detected by Cbp (green) and the apical surfaces by WGA (red). For gene knockdown n ≥ 15 individuals were analyzed and representative images are shown. While *wt* larvae showed a stratified chitin-matrix (red arrow), the individual *idgf1, idgf3, idgf4, idgf5* and *idgf6* knockdown led to deformed chitin-matrix morphology and narrowed cuticle thickness, as indicated by the white bars. White dashes mark the apical cell surface. (**e**) Obst-A was enriched at the apical epidermal cell surface (arrowheads) marking the cuticle assembly zone^16^, in *wt* as well as in *idgf1, idgf3, idgf4* knockdown larvae. Note that in RNAi-induced *idgf5* and *idgf6* mutants Obst-A was only partially enriched at the apical cell surface (arrowheads). Obst-A was additionally located in the lamellate procuticle of *wt* larvae (yellow arrow). Obst-A staining aggregated (blue arrows) in the procuticle in *idgf3, idgf5*, and *idgf6* mutant larvae; Obst-A was reduced upon *idgf1, idgf4* knockdown. Scale bars represent 10 μm.

Next we asked whether *Cht* and *idgf* genes are involved in providing epithelial barriers, which was analyzed in wounded RNAi knockdown larvae of first instar stage. Gentle lateral pricking of *wt* and *69B-Gal4* control larvae with a microinjection needle resulted in short hemolymph bleeding^16^,^19^ but did not critically impair the cuticle barrier since most animals survived the wounding. In contrast, UAS-*RNAi*-mediated knockdown of individual *Cht2, Cht5, Cht7, Cht9, Cht12* and of *idgf1, idgf3, idgf4, idgf5 and idgf6* genes displayed large epidermal lesions at wounded sites of first instar. Epidermal burst was followed by a severe hemolymph bleeding, organ spill out and immediate lethality (Figs 3a–c and 4a–c). Quantitative real-time PCR analyses confirmed high knockdown efficiencies for these *Cht* and *idgf* genes in first instar larvae (Supplementary Fig. S1e). In summary, these findings provide evidence that a large number of *Cht* and *idgf* genes are required at the epidermis for exoskeletal cuticle barrier formation.

The roles of individual *Cht2,5,7,9,12* and of *idgf1,3,4,5,6* genes in establishing cuticle barriers were further investigated by studying epidermal morphology. Third instar larvae deposit massive amounts of cuticle material to expand their number of chitin-lamellae within the procuticle^36^. The chitin-rich procuticle was visualized by the chitin binding probe (Cbp) and the apical cell surface was detected by the lectin Wheat germ agglutinin (WGA)^16^, which recognizes sugar residues of glycoproteins and chitin^37^. Confocal analysis of *wt* third instar larvae showed WGA enrichment at the apical cell surface and Cbp accumulation in the chitin-lamellae^16^. In *Cht* and *idgf* knockdown mutants the morphology of the epidermal cuticle was defective (Figs 3d and 4d). *Cht2* knockdown resulted in an extremely thin epidermal chitin-ECM. In *Cht5* RNAi-induced mutant larvae the lamellate procuticle structure was deformed. In *Cht7* knockdown larvae the chitin-ECM was disrupted and large blister-like cuticle extensions appeared. *Cht9* knockdown larvae showed a deformed and thin chitin-ECM. *Cht12* knockdown mutants revealed a thinned chitin-ECM and stratification defects. Our data of gene specific phenotypes provide first evidence that distinct *Chts* are required for epidermal cuticle morphogenesis. Gene expression profiles, individual domain variances (Supplementary Fig. S1) and enzyme specific activities against short or long polymeric chitin substrates^32^ suggest differences in Cht functions and thus further support our findings that several Chts are involved in organizing epidermal cuticles.

Analogous to *Chts* the knockdown of *idgfs* led to epidermal cuticle morphology defects. The histological analysis of third instar larvae showed unusually narrowed ECM thickness and deformed epidermal chitin-matrix in gene specific *idgf1,3,4,5,6* knockdown larvae. Cbp signals appeared reduced and characteristic lamellate chitin-scaffolds were not observed in the mutant larvae. These data indicate that the epidermal chitin-ECM was severely deformed in *idgf* knockdown larvae (Fig. 4d).

### *Chts* and *idgfs* coordinate chitin-scaffold and cuticle proteins for ECM formation at the apical cell surface

To understand the requirement of individual *Cht* and *idgf* genes in the molecular machinery of chitin-ECM formation, we studied if expression and localization of an essential cuticle component^16^,^19^ depends on the individual knockdowns. The chitin-binding protein Obstructor-A (Obst-A)^17^ coordinates dynamics of the growing chitin-scaffold^19^. Epidermal cells secrete Obst-A into the apical cuticle assembly zone, where it is required for the maturation of the chitin-ECM into a lamellar procuticle^16^. In addition, Obst-A is localized along chitin-lamellae of the procuticle (Figs 3e and 4e) where it is involved in chitin-matrix protection^16^. In contrast to *wt* epidermis, Obst-A enrichment in the cuticle assembly zone was disturbed in *Cht2,5*,*7,9*,*12* knockdown larvae (Fig. 3e). These findings show that *Chts* are required for Obst-A localization within the cuticle assembly zone which is important for chitin-ECM maturation at the apical cell surface^16^.

In contrast to *Chts*, knockdown of *idgf* genes did not disturb apical enrichment of Obst-A within the cuticle assembly zone (Fig. 4e). However, Obst-A was strongly reduced within the lamellate chitin-ECM in *idgf1*,*4*,*5,6* knockdown larvae and mislocalized in unusual plaque-like structures upon *idgf3* knockdown. These findings suggest that the non-chitinolytic Idgfs^26^ are involved in Obst-A-mediated protection of the chitin-ECM^16^.

### *Cht2* is required for ECM growth at the cuticle assembly zone

**Figure 5:**
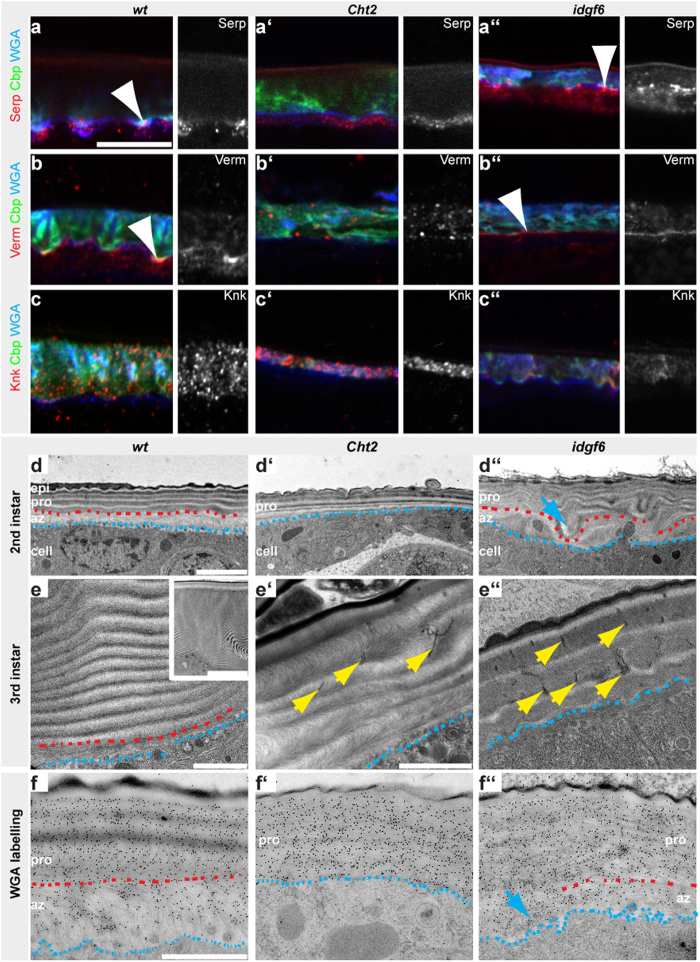
Cht2 and idgf6 are required for epidermal procuticle formation. Confocal analysis of cross sections of third instar larvae. (**a–b”**) Serp (**a**) and Verm (**b**) were enriched in the cuticle assembly zone of *wt* larval epidermis^16^. The *Cht2* knockdown larvae showed no apical Serp (**a’**) and Verm (**b’**) enrichment. Upon *idgf6* knockdown Serp (**a”**) and Verm (**b”**) were enriched at the apical cell surface (arrowheads). (**c–c”**) In *wt* larvae Knk was distributed in the procuticle at chitin-lamellae. In *Cht2* knockdown larvae Knk was normally distributed. Upon *idgf6* knockdown, Knk staining was affected when compared to *wt*. Scale bars represent 10 μm. (**d–d”**) Representative images show ultrastructure analysis of the larval body wall cuticle. For gene knockdown phenotypes of different larvae (*Cht2* RNAi n = 6 second instar; *idgf6* RNAi n = 5 second instar) were similar though variable in severity. The *wt* second instar (**d**) epidermal ECM contains the cuticle assembly zone (az), which was located at the apical cell surface, the lamellate chitinous procuticle (pro), the envelope, and the outermost epicuticle (epi). In *Cht2* knockdown larvae (**d’**) the cuticle assembly zone was absent and the procuticle contained irregularly spaced chitin-lamellae. The *idgf6* knockdown larvae (**d”**) contained the cuticle assembly zone, but irregular chitin-lamellae architecture (arrow) and defective separation of the epicuticle. Red dashes mark the border between az and pro; blue dashes indicate the apical cell membrane. (**e–e”**) In *wt* third instar (**e**) ECM the number of chitin-lamellae increases (overview in inset). Upon *Cht2* knockdown (n = 6; third instar) (**e’**) the number of chitin-lamellae was small. In *idgf6* RNAi mutants (n = 5; third instar) (**e”**) chitin-lamellae degraded. They show numerous electron dense pore canal-like structures (yellow arrows) which were distributed across the deformed procuticle. In *wt* third instar pore canal-like structures appeared restricted to the epicuticle and outermost procuticle lamellae (Fig S7). (**f–f”**) Immunogold-labeled WGA recognizes chitin^16^,^37^. In *wt* second instar WGA was enriched in the chitin-dense procuticle but was less often found in the cuticle assembly zone (**f**). In *Cht2* RNAi mutants (**f’**) the cuticle assembly zone was absent and WGA was distributed towards the apical cell surface. In *idgf6* knockdown larvae, WGA was enriched in the procuticle. WGA enrichment was partially found adjacent to the apical cell surface (arrow) which may reflect the wavy pattern of the procuticle (**d”**). Supplementary Fig. S3e shows the control. TEM studies n = 3; Scale bars represent 1 μm, in D–F”, and 2 μm in the inset.

Among *family18* members the *Cht2* and *idgf6* knockdown resulted in the most severe epidermal cuticle defects (Figs 1, 2, 3, 4) and highest lethality of larvae (Supplementary Fig. S1f,g). Since our data suggest different functions in cuticle formation for *Cht2* and *Idgf6* we wanted to molecularly analyze their roles when third instar larvae increase epidermal chitin-ECM size. Our data suggest that the molecular Obst-A network can be affected in the cuticle assembly zone by *Cht2* (Fig. 3e) and in the lamellate chitin-ECM by Idgf6 function (Fig. 4e). It was shown that Obst-A forms a core unit-complex with the deacetylases Serp, Verm^18^,^38^ and the GPI-linked Knk^39^ to promote chitin-matrix stability in trachea^19^. Epidermal cells secrete Obst-A into the cuticle assembly zone, where it is required for the localization of Serp and Verm in order to mature the chitin-matrix into a lamellar stratified ECM^16^. A second critical aspect for exoskeletal barrier formation is the Knk-mediated chitin-ECM stability^20^,^21^ and its putative role against degradation in *Drosophila* which involves Obst-A at the chitin-lamellae^16^. Defects either in expression levels or extracellular localization of these proteins lead to severe larval cuticle defects^16^ which we found in *Cht2* and *idgf6* knockdown larvae (Figs 3 and 4). We therefore addressed whether *Cht2* or *idgf6* have any influence in expression and localization of the Obst-A cuticle partners Serp, Verm and Knk. In *wt* third instar larvae the Serp and Verm deacetylases were enriched at the epidermal apical cell surface. In contrast, apical enrichment of both appeared disturbed in the *Cht2* knockdown mutants. Confocal studies revealed reduced extracellular Serp levels and mislocalization of Verm (Fig. 5a,a’). These findings suggest that *Cht2* is involved in Serp and Verm-mediated chitin-matrix maturation, providing essential chitin-ECM stability^18^,^38^. We then asked whether *Cht2* could additionally play a role in protection of the established ECM. It was shown that Knk is involved in defending the newly formed chitin-matrix against chitinolytic degradation in the *Tribolium* body wall cuticle^20^ which could be also the case in the growing epidermal procuticle of *Drosophila* third instar larvae^16^. However, *wt* and *Cht2* knockdown larvae showed similar Knk localization pattern indicating that Knk-mediated cuticle function appeared unaffected (Fig. 5c,c’). Our localization data propose that *Cht2* activity affects the cuticle assembly zone machinery to organize and mature the chitin-matrix in third instar larvae at intermolt stage.

In order to verify whether the cuticle assembly zone is defective in the *Cht2* knockdown we addressed epidermal ECM histology by ultrastructure analysis (Fig. 5d,d’). In *wt* larvae the chitin-ECM of the cuticle is represented by the cuticle assembly zone, next to the the apical cell surface, and the outer lamellate procuticle (Fig. 5d). In third instar larvae procuticle size starts to increase dramatically by producing one chitin-lamella per hour^36^,^40^. This leads to a multiple number of chitin-lamellae at the end of larval development (Fig. 5e). In *Cht2* knockdown larvae the assembly zone was barely detectable or even absent. Procuticle lamellae appeared irregularly stratified and, moreover, the number of procuticle lamellae did not increase during intermolt of third instar larvae (Fig. 5d’), likely causing the narrowed ECM size in these larvae (Fig. 3d). The absence of the cuticle assembly zone was further verified by immunogold-labelled WGA, which recognizes tightly packed chitin. In the *wt* epidermis numerous WGA-gold conjugates accumulated in the chitin-rich lamellate procuticle but not in the assembly zone. In contrast, in *Cht2* knockdown larvae such differences in WGA distribution were not detected (Fig. 5f–f’). The unusual WGA accumulation towards the apical cell surface indicates strong reduction or loss of the cuticle assembly zone in *Cht2* mutants.

### *idgf6* is essential for cuticle maintenance

In sharp contrast to *Cht2* mutants, knockdown of the *idgf6* gene did not disturb enrichment of Obst-A, Serp and Verm at the apical cell surface within the cuticle assembly zone (Figs 4e and 5a–b”). Upon *idgf6* knockdown Knk was localized within in the cuticle but normal extracellular accumulation appeared affected when compared to *wt* (Fig. 5c,c”). This suggests that potentially chitin protection could be altered, and this may cause a premature degradation of the lamellate procuticle^16^,^20^,^21^.

Analogous to the *Cht2* mutant ultrastructure analysis we investigated the epidermal cuticle in *idgf6* mutant larvae. In second instar larvae *idgf6* knockdown led to unusually wrinkled epidermal procuticle morphology which partially affected the cuticle assembly zone (Fig. 5d”). In third instar, the epidermal procuticle appeared degraded (Fig. 5e”) and its thickening by adding numerous chitin-lamellae was disturbed, finally causing the narrowed cuticle size. The premature procuticle degradation may have further consequences. In insects pore canals transport material across the procuticle^41^,^42^,^43^,^44^. In *Drosophila* pore canals are found in the procuticle where they expand to form a lumen^15^. In *wt* second instar we identified pore canal-like structures running across the procuticle. The *wt* third instar showed pore canal-like structures only in the epicuticle and the outermost two chitin-lamellae of the procuticle (Supplementary Fig. S3). In contrast, upon depletion of *idgf6* in third instar larvae, numerous unusually electron dense pore canal-like structures were found in the deformed procuticle (Fig. 5e”; Supplementary Fig. S3). Degradation of the procuticle and defective structure of pore canals may interfere with outer cuticle layers since the epicuticle was irregularly and not clearly separated from the underlying procuticle in *idgf6* knockdown larvae. Altogether, this implies that the chitinolytically inactive Idgf6^32^ may contribute to the stability and protection of the growing procuticle.

### *Cht2 and idgf6* function is essential for epithelial defense against pathogens

**Figure 6:**
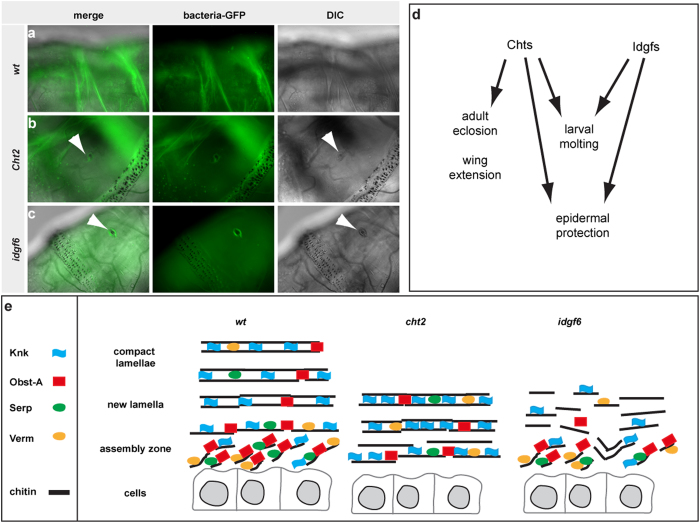
The developmental roles of the *Cht*- and *idgf* family. (**a–c**) GFP expressing *Pseudomonas entomophila* usually cannot penetrate through the cuticle barrier of the *wt* instar larva (n = 45). For most *Cht2* and *idgf6* knockdown (each n = 45) larvae *Pseudomonas* penetrate the defective cuticle barrier for invasion resulting in immune triggered melanization-like darkening spots at the wound surface (arrowheads). Normarski-optic reveals darkening of cuticle material which was attacked by invading bacteria. (**d**) The drawing scheme summarizes phenotypic data resulting from knockdown of *Chts* and *idgf* genes. (**e**) The schematic model summarizes our findings in size regulation of the epidermal cuticle in third instar larvae, which requires core cuticle components such as Obst-A, Serp and Verm enriched in the cuticle assembly zone to mature the chitin-lamellae^16^. It further requires the Knk protein, which has an important role in protecting the newly synthesized cuticle in *Tribolium*, and potentially also in *Drosophila*^16^,^20^. Our data provide evidence that in *Cht2* knockdown larvae the cuticle assembly zone is absent and core cuticle components required for maturation are mislocalized, finally resulting in reduced ECM thickness. In *idgf6* RNAi mutants, chitin-lamellae are degraded and localization of procuticle components is disturbed causing a defective cuticle.

Cuticles continuously withstand numerous environmental physical and chemical traumas, such as infections by invading microorganisms or harmful and toxic reagents. We addressed the *in vivo* consequence of defective cuticle barriers in protecting RNAi knockdown animals against invasive microorganisms. GFP expressing *Pseudomonas entomophila* normally do not penetrate the epidermal cuticle but infect *Drosophila* larvae orally leading to damage of the gut epithelia which causes melanization spots in the wounds^45^. *Pseudomonas* successfully penetrated the defective cuticles of *Cht2* and *idgf6* knockdown larvae. With the exception of one animal all *wt* larvae (n = 45) were not penetrated by *Pseudomonas* at the epidermal cuticle, as GFP spots and the accompanied melanization darkening were not observed. In contrast, most of the *Cht2* and *idgf6* RNAi induced mutants (n = 45 for each genotype) showed melanization-like darkening spots at the epidermal wounding sites where GFP-expressing bacteria accumulated (Fig. 6a–c). These results indicate the fragility of the outermost physical defense barrier in the mutants and show that *Cht2* and Idgf6 are critical for the susceptibility of the body wall cuticle to invading microorganisms.

## Discussion

We investigated the role of *Drosophila family18* genes in organizing cuticle molting and barrier protection (Fig. 6d). Our data are in line with previous observations^26^,^46^. It was recently shown in the brown plant hopper *Nilaparvata lugens* that double stranded RNA injection directed against *Cht1, Cht5, Cht7, Cht9* and *Cht10* causes mortality^46^. We observed larval and pupal lethality upon knockdown of the individual of *family18* members. The *Cht5* gene knockdown caused pupal molting defects in *Tribolium* similar to our study. Findings that the *Cht10* (encoded by the *Drosophila* Cht1/3/10 gene) RNAi-induced knockdown showed lethality and severe cuticle molting defects in larvae and pupa of the red flour beetles^47^ supplements our knockdown screen for *Chts* in *Drosophila*. The observation that *Drosophila Cht11* did not cause any molting or barrier defects is consistent with its recently proposed function in the mitochondria^48^. The *Cht4* knockdown did not show any cuticle phenotypes in *Drosophila* and other insects, but *Cht4* is rather involved in reproduction^46^. However, previous data indicated functional specialization among *Cht* genes for insect molting^26^. Our systematic study in *Drosophila* extends initial observations and shows that a large number of *Cht* and *idgf* genes are required to control the repetitive reorganization of the chitin-ECM at the epidermis.

Moreover, our screen characterizes which *Cht* and *idgf* genes are essential for forming the epidermal barrier-ECM. We show that the *Cht2,5,7,9,12* and *idgf1,3,4,5,6* genes are required for chitin-ECM formation and barrier function throughout larval development. These morphological data and *in vivo* barrier analysis were supported by analysis of the cuticle structure and the involved molecular network. The cuticle assembly zone is associated with the apical cell surface^49^ where chitin-fibrils and associated proteins arise. We recently showed that apically secreted components are organized within the assembly zone into the functional ECM matrix^16^. Here we provide evidence that *Cht2* is required for cuticle assembly zone function. In the *Cht2* knockdown the assembly zone was absent and consequently Obst-A, Serp and Verm were not enriched at the apical cell surface but mislocalized or reduced. This finally led to reduced number and defective morphology of chitin-lamellae when normally the number of lamellae should increase in third instar and the cuticle becomes thickened. There are different molecular scenarios how the glycosylhydrolase *Cht2* could determine procuticle size. It could act in the cuticle assembly zone in order to modify for example length of chitin-fibrils, which supports Obst-A-mediated chitin-scaffold formation and maturation by the deacetylases Serp and Verm. A distinct scenario could be that *Cht2* inhibits premature assembly, or acts in later stages for elongation and ordering the filaments. Defects in each of these processes could lead to pushing the cuticle proteins towards the membrane, and the disappearance of cuticle assembly zone. Indeed, preliminary data for *Cht2* protein localization within the epidermal cuticle support both scenarios (Supplementary Fig. S4).

Recently, it has been shown in *Tribolium* that downregulation of *Tc-Cht5* and *Tc-Cht10* along with *Tc-knk* rescued the *knk* mutant phenotype and restored chitin levels but not laminar architecture. The single and even double knockdown of *Tc-Cht5* and *Tc-Cht10* did not disturb laminar organization of the cuticle^20^. Based on our finding, we suggest that Chts may have different roles in organizing cuticle molting and cuticle formation. For example, the *Drosophila Cht5* knockdown showed partially enriched Obst-A at the assembly zone and only minor reduction of cuticle thickening. Whether it plays a role in Knk turnover at the old cuticle as found for *Tc-Cht5* remains elusive^20^. In contrast, *Cht2* knockdown did not affect Knk localization or levels, but led to the absence of assembly zone formation and mis-localization of associated molecular modifiers resulting in a lamellar architecture but cuticle thickening was disturbed. Furthermore, in contrast to *Cht2*, in *idgf6* mutants the procuticle was degraded, showing rudimentary chitin-lamellae and an irregularly and not clearly separated epicuticle. Reduced *idgf6* did not disturb the assembly zone and its modifiers Obst-A, Serp and Verm at the apical cell surface but interfered with normal Knk levels. Altogether, this implies that the chitinolytically inactive Idgf6 (synonymous to Cht13 and DmDS47)^32^ may contribute to the stability and preservation of the growing procuticle. Similar phenotypes have been found upon *Tc-Knk* downregulation at the body wall and elytra cuticle^20^. Whether Idgf6 binds chitin for rather direct protection or it may act indirectly via potential Knk function is not known.

Although gene expression patterns are common among essential *Cht* genes (Supplementary Fig. S2c–j), individual domain arrangements (Supplementary Fig. S1), such as chitin-binding and transmembrane domains, and in case of *Cht5 and Cht9* enzymatic specificity for substrates and for pH profile^25^,^31^, support our studies and suggest that a large variety of different glyco-18 domain hydrolases could be required for modulating the ECM in the growing cuticle at the same time. Essentially, also *idgf* genes show a common expression pattern in the cuticle producing organs, but their gene-products are most likely enzymatically inactive, as shown for the *Drosophila* Idgf6 and the *Tribolium* IDGF2 and IDGF4^32^. This group of proteins likely binds to chitin^25^ and may thereby contribute to the protection of the ECM.

Our study demonstrates that *Cht2* and *idgf6* are very critical for larval cuticle barrier formation (Fig. 6e) and protection against invasive microorganisms and mechanical stresses. *Cht2* and *Idgf6* genes offer new targets for a gene specific search of insecticides, since the epithelial barrier was impaired and resulted in a premature lethality of animals and a higher susceptibility to pathogenic infections. Homologues of the *family18* genes exist in parasites and pest harmful to mankind and agriculture, such as mosquitos, lice and the spotted wing *Drosophila*^50^,^51^,^52^. Blast searches showed that parts of the C-terminus of *Cht2* and the N-terminus of Idgf6 were not conserved among the insects providing a specific target for insecticides (Supplementary Fig. S5). This opens the possibility to search for small molecules that may inhibit function of insect species specific *Cht2* or Idgf6 without affecting beneficial organisms. Chitin-ECM is the most important insect barrier against any environmental stresses. Affecting function of hydrolase enzymes and the ECM protection system provide new strategies for eliminating problematic animals already during larval stages and preventing the growth of next generations of upcoming pest.

## Methods

### Fly stocks

Vienna (Austria) /Bloomington (USA) stock centers (http://stockcenter.vdrc.at/control/main and http://flystocks.bio.indiana.edu/): *w1118* (referred to as *wild-type (wt)*), *69-BGal4* line, UAS-*RNAi*-*Cht* and UAS*-RNAi-idgf* lines (Vienna stock center). For knockdown analysis, *69B-Gal4* flies were mated with UAS**-RNAi flies. For negative controls we used *Wild-type (wt)* animals and animals from the *69B-Gal4* and the different UAS-*RNAi* lines but without inducing the RNAi system. In general, RNAi induced phenotypes can be variable to some extent. We therefore show representative images of observed phenotypes and indicate the number of analyzed animals.

### Lethality and cuticle assays

Embryos from *wt, 69B-Gal4* stocks and from individual *69B-Gal4* driven UAS-*RNAi cht*/*idgf* crosses were grown on apple juice agar plates at 29 °C for until they reached end of embryogenesis. After dechorionation with 2.5% sodium hypochlorite for 5 minutes, living stage (st) 17 embryos (n ≥ 100 for Chts n ≥ 75 for idgfs) and were transferred onto new agar plates and incubated at 29 °C. The number of dead and living animals were counted every 24 hours with Stemi stereomicroscopes (Zeiss, Germany) and characterized from first instar larvae to adult flies. Every genetic experiment was repeated at least three times. For cuticle preparation first, second and third instar larvae were washed with water and incubated in a 1:1 lactate and Hoyer’s reagent mixture overnight at 65 °C. Larval stages were determined by the morphology of mouth hooks. Larvae were analyzed with 60x and 100x objectives in Olympus immersion oil and by Normarski microscopy (DIC; Olympus AX70). Pupae and adult flies were analyzed using an Olympus Binocular microscope (SZX12). All images were taken with Cell’F software. In general for all assays (see below) images were cropped in Adobe Photoshop CS6 and figures designed with Adobe Illustrator CS6. For statistical analysis Excel 2010 was used.

### Epithelial cuticle/epidermis integrity assay

First instar larvae (n > 50) were gently laterally wounded with a standard glass needle for micro injections as recently described^16^,^19^. The integrity test was repeated at least three times each with a minimum of thirty animals and immediately monitored by Normarski microscopy (DIC; Olympus AX70) and Cell’F software.

### Larval fixation and immunofluorescence studies

Third instar larvae were fixed overnight in 4% paraformaldehyde and embedded in JB-4 resin (Polysciences, USA, Warrington, USA) as described by the manufacturer. Slices of 7 μm were cut using an Ultracut E (Reichert-Jung, Solms, Germany). For each genotype n ≥ 15 individuals were analyzed. The immunofluorescent labeling was performed as recently described^16^. For labeling chitin we used Alexa488-conjugated chitin-binding-probe (Cbp, 1:50, New England BioLabs, USA). For detecting the apical cell surfaces we used Alexa633-conjugated wheat germ agglutinin (WGA, 1:250; Molecular Probes, Carlsbad, USA). The WGA selectively detects *N*-acetylneuraminacid and *N*-acetylglucosamine sugar residues and can be used as a marker for plasma membrane surfaces and cuticle layers. Used antibodies: Knk (1:333; rabbit)^39^, Obst-A (1:300; rabbit)^19^, Serp (1:175; rabbit) and Verm (1:175; rabbit)^18^. Secondary antibodies were linked with fluorescent dyes (Dianova, Hamburg, Germany and JacksonImmuno, Baltimore, USA). The samples were mounted in Vectashield (H1500, DAPI, Vector Laboratories, Burlingame, USA).

For immunofluorescent analysis LSM710 and LSM780 (Zeiss, Jena, Germany) confocal microscopes and 63x LCI Plan Neofluar objectives were used. For scanning procedure pinhole “airy unit 1” ZEN software standard settings were used and cross-talk was avoided by using according filter settings and lasers. Images were taken with the ZEN 2010 and ZEN 2011, cropped with Adobe Photoshop CS6 and figures were designed with Adobe Illustrator CS6.

### Ultrastructure analysis

*Drosophila* second and third instar larvae were placed on a 150 μm flat embedding specimen holder (Engineering Office M. Wohlwend GmbH, Sennwald, Switzerland) and frozen using 1-hexadecene in a Leica HBM 100 high pressure freezer (Leica Microsystems, Germany) according to the described method^16^. Wheat Germ Agglutinin (5 μg/ml, Vector Labs Burlingame, USA) was detected by a rabbit-anti-biotin antibody (3.9 μg/ml, Rockland, USA) and Protein A gold (10 nm, 1:100, G. Posthuma, Utrecht, Netherlands) as described recently^16^. Images were taken by a Philips CM120 electron microscope (Philips Inc.) using a TemCam 224A slow scan CCD camera (TVIPS, Gauting, Germany).

### Infection assay

*Pseudomonas entomophila (PE)* was cultured in LB-medium with 50 μg/ml rifampicin at 29 °C. For infection experiments, second instar larvae were placed on filter papers moistened with PBS. For each genotype and condition, three dishes were prepared. Uninfected control larvae were fed with 500 μl saccharose solution. For infection of larvae, 500 μl of 10% saccharose solution containing 0.5 g resuspended bacteria was added to the filter paper. Larvae were incubated at 29 °C for 24 h and afterwards imaged with a Zeiss Apotome Axiovert 200 M (25x Plan Neofluar objective; Zeiss, Jena, Germany). *Pseudomonas* positive larvae reveal the GFP signal. Multidimensional photographs were taken with AxioVision software (Zeiss). For each genotype n = 45 animals were analyzed and the infection experiment was repeated three times. To prove infection by *Pseudomonas* and to rule out contamination of the control, homogenized larval material was streaked out on Rifampicin-agar plates with milk, on which pathogenic *Pseudomonas* grows colonies. After overnight culturing at 29 °C only plates with infected animals showed *Pseudomonas* colonies, while non-infected did not.

### Quantitative real-time PCR (qPCR)^53^

To test RNAi kockdown efficiency L1 larvae were used. Samples were collected into lysis buffer with ß-Mercapoethanol (Roth, Karlsruhe, Germany) and lysed in a Precellys homogenizer using a 1.25–1.65 diameter glass bead mix (Roth, Karlsruhe, Germany). The lysed samples were frozen in liquid nitrogen and stored at −80 °C. RNA was extracted within two weeks. The NucleoSpin RNA II Kit (Macherey-Nagel, Düren, Germany) was used following the manual instructions including DNase treatment. RNA quantification was performed by the use of NanoDrop (ThermoScientific, Waltham, USA). A second genomic DNA digestion and the first strand cDNA synthesis was carried out with 500 ng of total RNA using the QuantiTect Reverse Transcription Kit (Qiagen, Hilden, Germany) following the manual instructions and using the provided oligo-dT and random primer mix. All RNA reactions were performed on ice, cDNA was stored at −20 °C. qPCR experiments were done as recently described^54^ with the iQ5 Real-Time PCR detection system (Bio-Rad, München, Germany). qPCR primers have been designed with the “primer3plus” software using standard adjustments for qPCR primers leading to an amplicon length between 75 and 150 nucleotides (manufactured by Invitrogen, Karlsruhe, Germany). Desalted qPCR primers have been manufactured by Invitrogen (Karlsruhe, Germany). For PCR reaction we used 1 μl cDNA, primers in a final concentration of 200 nM and the iQ SYBR Green Supermix (Bio-Rad, Munich, Germany) in a total volume of 25 μl. Reactions were performed as technical duplicates or triplicates in clear 96-well qPCR plates (Bioplastics, Landgraaf, Nederlands). For normalization of transcript levels we used *rp49* (Ribosomal protein L32) as a reference. The specificity of all primers was tested by melt curve analysis. Standard control PCR reactions (no-template control; negative control) were performed and showed no signal. Expression data were calculated according to the delta-delta-CT method and analyzed using Bio-Rad iQ5 Optical System Software and Microsoft Excel 2010. All primer sequences and efficiencies are provided for *Chts* (Supplementary Table S1), for *idgfs* (Supplementary Table S2) and the FlyBase gene card web-links are given in (Supplementary Table S3).

## Additional Information

**How to cite this article**: Pesch, Y.-Y. *et al*. Chitinases and Imaginal disc growth factors organize the extracellular matrix formation at barrier tissues in insects. *Sci. Rep*. **6**, 18340; doi: 10.1038/srep18340 (2016).

## Supplementary Material

Supplementary Information
