# Supplementary material for: Chitinases and Imaginal disc growth factors organize the extracellular matrix formation at barrier tissues in insects
Source: Sci Rep. 2016 Feb 3;6:18340. doi: 10.1038/srep18340 (PMC4738247; doi:10.1038/srep18340)
Supplement: Supplementary Information [file srep18340-s1.pdf]

Supplementary Information

Chitinases and Imaginal disc growth factors organize the extracellular matrix formation at barrier tissues in insects

Yanina-Yasmin Pesch[a,b], Dietmar Riedel[c], Kapil R Patil[b], Gerrit Loch[b], and Matthias Behr[a,b]*

[a]Institute for Biology and Translational Center for Regenerative Medicine (TRM), University of Leipzig, 04103 Leipzig, Germany

[b]Life & Medical Sciences Institute (LIMES), University of Bonn, 53115 Bonn, Germany

[c]Max Planck Institute for Biophysical Chemistry, Electron microscopy group, 37077 Göttingen, Germany

*Correspondence to: matthias.behr@uni-leipzig.de

Supplementary Figures and Legends

Supplementary Fig. S1

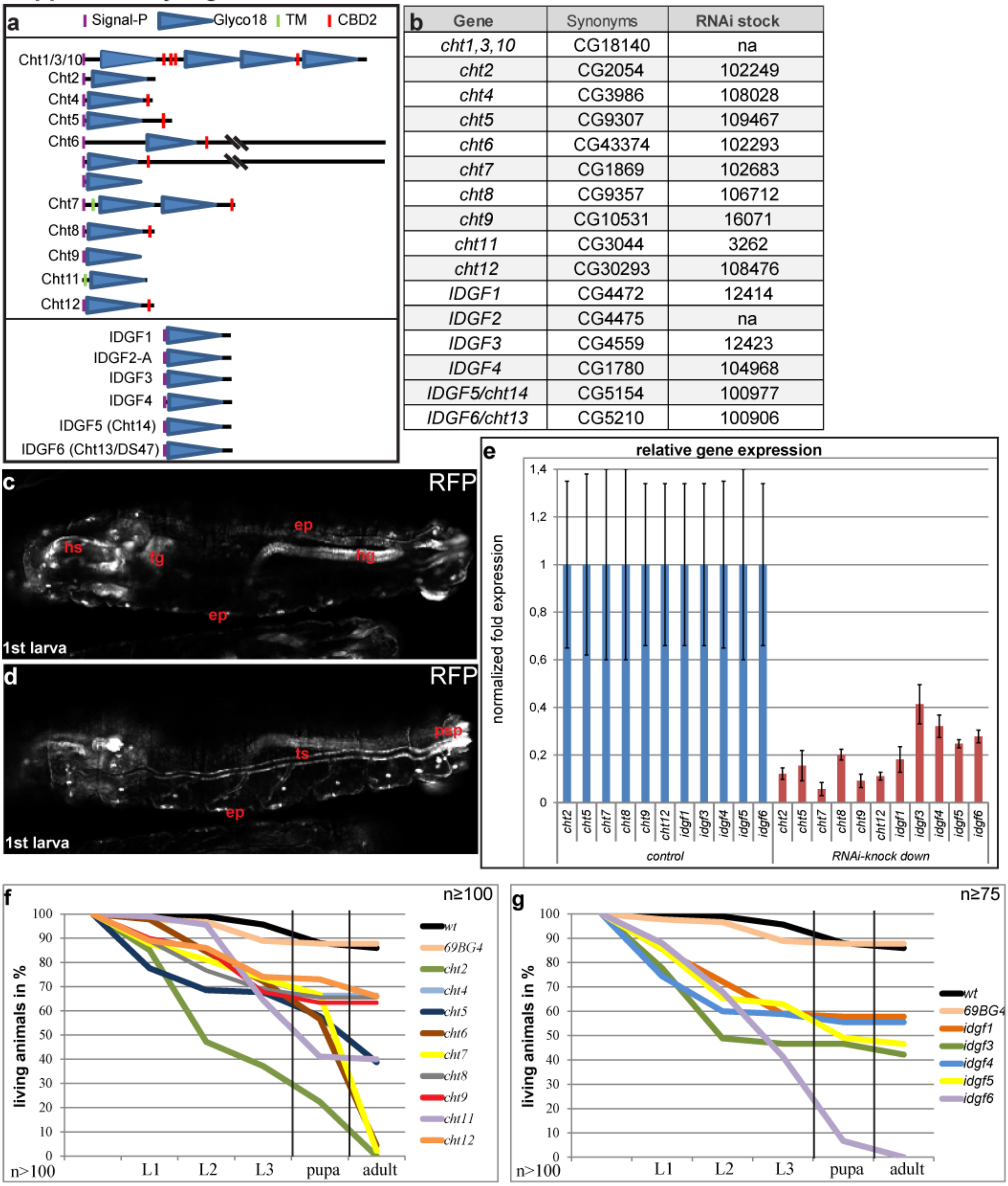

b Gene	Synonyms	RNAi stock
cht1,3,10	CG18140	na
cht2	CG2054	102249
cht4	CG3986	108028
cht5	CG9307	109467
cht6	CG43374	102293
cht7	CG1869	102683
cht8	CG9357	106712
cht9	CG10531	16071
cht11	CG3044	3262
cht12	CG30293	108476
IDGF1	CG4472	12414
IDGF2	CG4475	na
IDGF3	CG4559	12423
IDGF4	CG1780	104968
IDGF5/cht14	CG5154	100977
IDGF6/cht13	CG5210	100906

The *Drosophila family18* members, used RNAi stocks and knockdown expression effects.
(a) Schematic drawings of the glycosylhydrolase *family18* indicate the Glyco18 domains (blue), N-terminal Signal-Peptides (Signal-P, purple), transmembrane domains (TM, green) and chitin-binding domains (CBD2, red). The *Drosophila family18* contains ten genes encoding the Chitinase (Cht) and six genes encoding the Imaginal disks growth factor (Idgf) proteins. The diagram illustrates that Chts contain individual and Idgfs common domain arrangements. Cht1, 3 and 10 are

encoded by a single gene. For Cht6 different protein variants were predicted. Domains were annotated with the SMART (EMBL) database and the CBS Server (CBS: http://www.cbs.dtu.dk/) (SignalP 4.1). **(b)** The table summarizes the *Drosophila family18 cht* and *idgf* genes. The FlyBase gene accession numbers and used Vienna RNAi stocks are provided. With the exception of *Cht1/3/10* gene and the *idgf2* gene, RNAi knockdown lines were used for all other genes. However both genes were investigated previously in *Tribolium castaneum*. In particular the *Cht1/3/10* gene showed severe cuticle molting defects throughout development [1]. These data supplement our knockdown screen. It is of note that the knockdown strategy with Vienna RNAi stocks resulted in efficient reduction of transcript levels. **(c,d)** The *69B-Gal4* drives embryonic and larval RFP expression in cuticle forming epithelia such as the epidermis (ep), head skeleton (hs), parts of the digestive system (foregut, fg; hindgut, hg), posterior spiracles (psp) and tracheal system (ts). **(e)** The quantitative RT-PCR analysis compares relative expression levels of *Chts* between *wt* and knockdown first instar larvae. Progeny of crosses from individual UAS-RNAi lines with *69B-Gal4* which showed severe epidermal cuticle defects (Figures 1,2) and reduction of expression levels upon *Cht2, Cht5, Cht7, Cht9* and *Cht12* as well as *idgf1, idgf3, idgf4, idgf5,* idgf6 knockdown. Standard deviation bars are indicated. The analysis shows fold expression levels normalized to *wt* (1 = 100%). **(f,g)** Lethality of animals upon UAS-RNAi-mediated knockdown of *Cht* (f) and *idgf* (g) genes using the *69B-Gal4* driver line. Individual knockdown animals (n≥100) were monitored for twelve days after egg laying, which is an adequate time period to analyze embryogenesis, larval stages, pupal development and early adulthood. In summary, this assay showed lethality during larval and pupal stages for individual knockdown of all tested *Drosophila Chts*/*idgfs*, with variability in the phenotypical severity, while *wt* and *69B-Gal4* control animals survived until adulthood. The highest lethality was caused by the *Cht2* and *idgf6* knockdowns during larval stages.

Supplementary Fig. S2

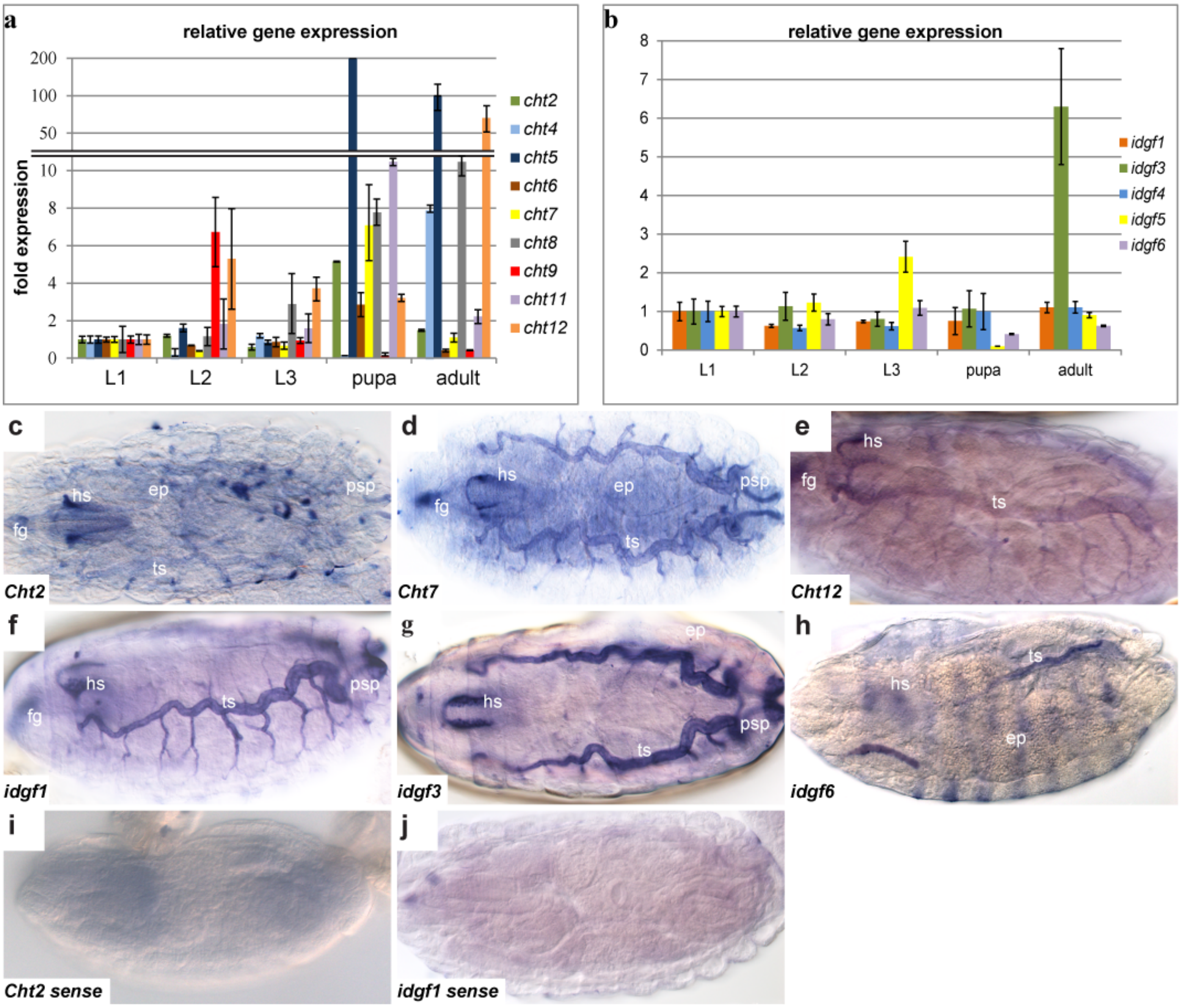

Spatial-temporal gene expression profiles of *Cht* and *idgf* genes.

(a, b) Transcription levels of *chitinase* (a) and *idgf* (b) genes relative to first instar larvae. Most *Cht* genes are constantly expressed throughout larval stages. The *Cht9* gene is up-regulated at second instar, which matches its specific requirement for molting into third instar. The *Cht12* gene is more strongly expressed during larval stages and mutants showed only larval molting defects. The *Cht2,5,6,7* genes show strong up-regulation during pupal stages reflecting their involvement in pupal molting. Note that Cht5 expression was > 323 fold upregulated in pupae. It is of note that *Cht8* and *Cht11* gene expressions are enriched during pupal development, but pupal molting defects were not observed. The *Cht11* gene product was discussed to be associated with mitochondrial functions for identification of critical infections [2]. The function of *Cht8* remains unknown. *Chts5,8,12* and *idgf3* are specifically enriched in adult flies, and may take over their enzymatic function later on after development. **(c-j)** Stage 17 whole mount embryos were stained by *in situ* hybridization as previously described [3] with DIG-labeled RNA antisense (c-h) and sense (i,j) probes. The *Cht2, Cht7, Cht12, idgf1, idgf3*, *idgf6* genes show mRNA expression in the cuticle producing organs, the mouth hooks (mh), the epidermis (ep), the tracheal system (ts) and the posterior spiracles (psp). Little expression is also detected in the foregut (fg). In contrast, the negative controls, the sense probes, did not detect expression.

Supplementary Fig. S3

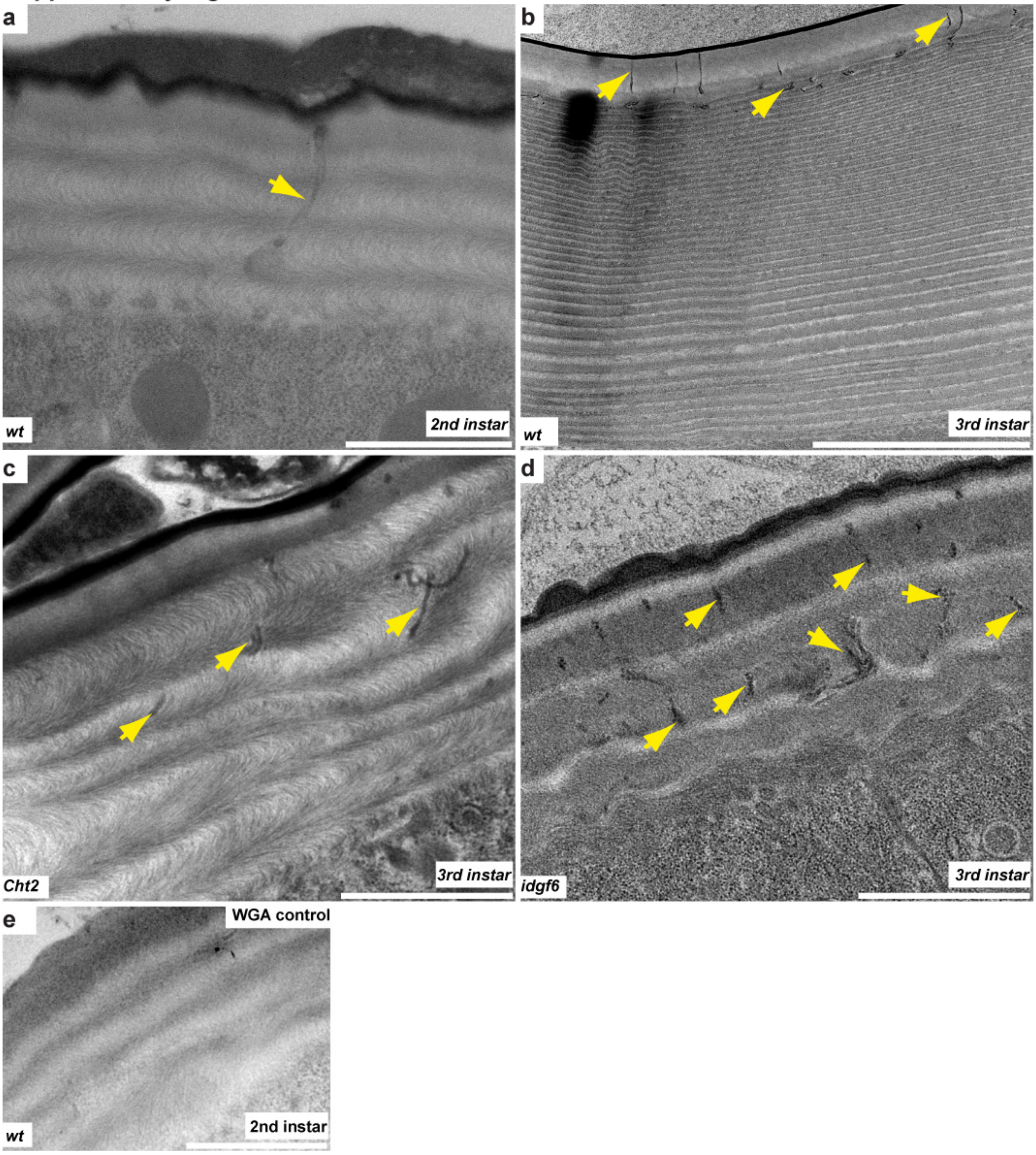

Epidermal cuticle formation in second and third instar larvae.
Ultrastructure analysis of epidermal cuticle in third instar larvae. **(a,b)** In *wt* epidermis the cuticle contains pore-canal-like structures (arrows). The second instar (a) shows canal-like structures running across the procuticle. The third instar (b) shows pore-canal-like structures only in the epicuticle and the outermost two chitin-lamellae of the procuticle. **(c)** In *Cht2* knockdown larvae, pore-canal-like structures appeared in the procuticle but did not cross the lamellae. **(d)** In the *idgf6* knockdown epidermis, pore-canal-like structures (dashes) are distributed throughout the degraded procuticle. **(e)** Negative control for WGA labeling shown in Fig. 5f-f''. Second instar larvae after immunogold-labeling but without using WGA. Scale bars represent 5 µm in B and 1 µm in a,c,d,e.

Supplementary Fig. S4

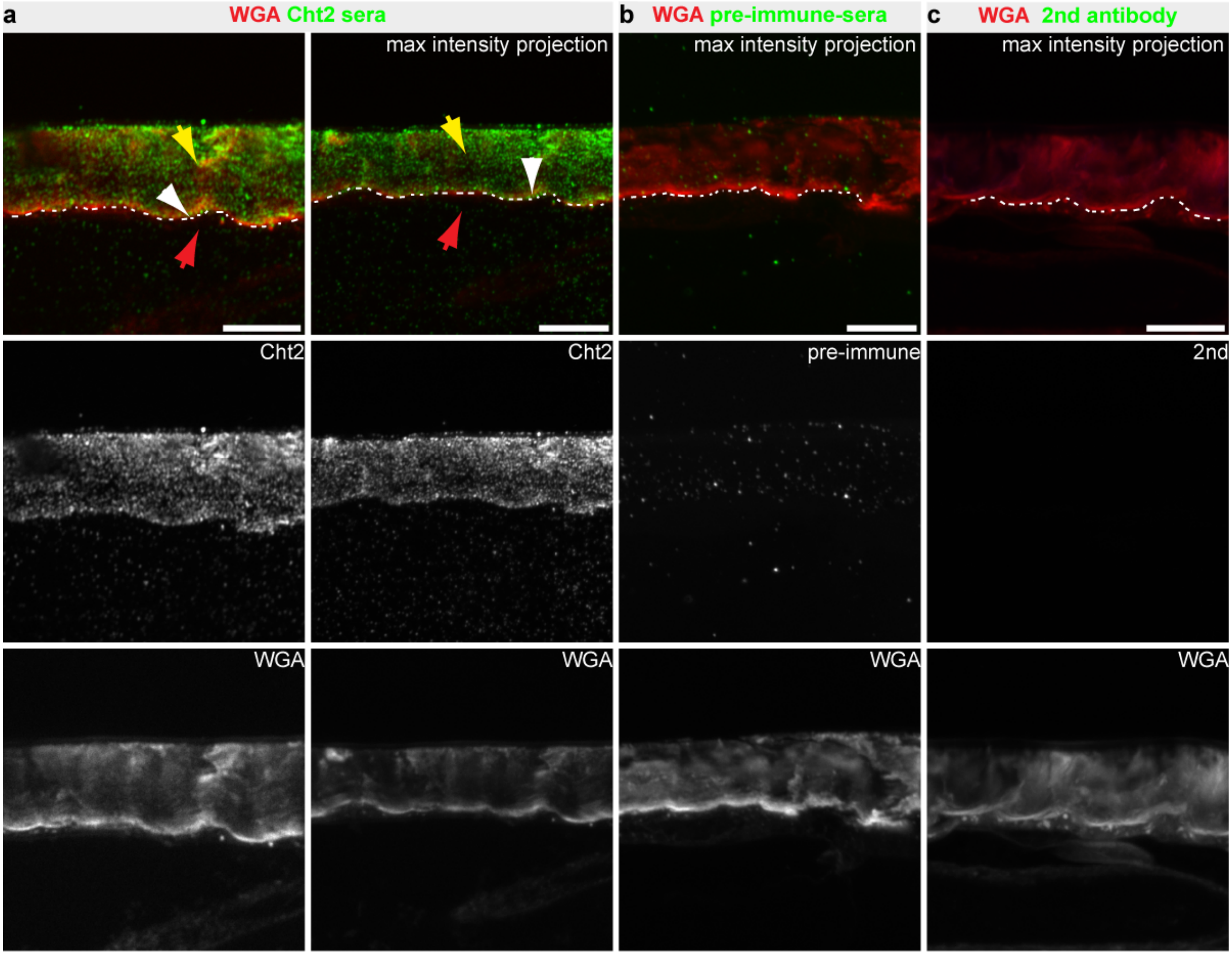

Cht2 is localized within the larval cuticle.

Immunofluorescent detection of Cht2 in the cuticle of wt third instar larvae by using Cht2 antibody serum and an anti-rabbit Cy-3 secondary antibody. The chitin-matrix and the apical surfaces were visualized by WGA (red). **(a,b)** Co-labeling studies (n=7 larvae) were performed with the Cht2 antibody serum (rabbit, green) shown as single image (a) or as confocal Z-stack projection (b). **(c,d)** As negative controls (n=7 each) the pre-immune serum (c) and the Cy-3 secondary antibody alone were used (d). Our preliminary data show representative Cht2 staining (a,b) within the cuticle (yellow arrow) and at the apical cell surface where the assembly zone is expected (white arrows). The epidermal cells underneath are indicated by the red arrows. The negative controls reveal no or very little signal in the cuticle (c, d). For comparison, all confocal settings were adjusted to similar settings (laser power, available gain functions, off set, pinhole size). Anti-Cht2 antibody was generated in rabbits (Pineda, Germany) against the peptide WDPQTSQVLAKSERN (amino acid 335 to 349). The peptide sequence is specific to all predicted Cht2 protein variants (see FlyBase) and did not show any cross-reactivity to other *Drosophila* proteins as tested by FlyBase and NCBI Blast search engines with stringent and less stringent matrices. For stainings the unpurified Cht2 antibody serum was used in a 1:1000 dilution. Contrast and brightness of images of original data were not changed afterwards. White dashes mark the apical cell surface. Scale bars indicate 10 µm.

Supplementary Fig. S5

a

Aedes aegypti (Aa) — *Drosophila melanogaster (Dm)*

```
DmCht2  39   HDKVVVCYVSTWAVYRPEQGAYAIENFDPNLCTHVVYAFAGLDITQAAIKSLDPWQDLKE  98
             H +VVVCY+S+WAVYR   G+++++NFDPNLCTHVV+A+AGLD+ +  IKSLD WQDLK+
AaCht2  8    HGRVVVCYISSWAVYRSGAGSFSLKNFDPNLCTHVVHAYAGLDVERNTIKSLDRWQDLKD  67

DmCht2  99   EYGKGGYEKMTGLKRSHPHLKVSLAIGGWNEGSANYSTLVANNLLRGRFVKQVSSFIRKY  158
             YG GGYEK+  L         + +++GGWNEGS  YS L A+   R  F +   +FIR+Y
AaCht2  68   NYGLGGYEKLVNL--------LMISMGGWNEGSVKYSKLAASQQRRQVFAENALNFIRRY  119

DmCht2  159  NFDGLDLDWEYPTQRKGKPADRENFVLLTKELREEFDEHGLLLTSAIGASKKVIDEAYDV  218
             FDGLDLDWEYPTQR G+P DRENFV L +EL ++F ++ LLLTS I A + VID AYD+
AaCht2  120  GFDGLDLDWEYPTQRGGEPNDRENFVQLVRELSQKFKKNNLLLTSTIEAKQYVIDAAYDI  179

DmCht2  219  RQISRYLDYLHIMCYDYHGSWDRRVGYNAPLTAPADDPLSVKFSIDYLLKLGAPPEKLVM  278
              +++YLD LH+ CYDY G W++++G+NAPLT   D+  +V+F+I++LL LGAPP KLV+
AaCht2  180  ENLTKYLDLLHVKCYDYRGIWNQKIGFNAPLT--GDEVHNVEFTIEHLLALGAPPNKLVL  237

DmCht2  279  GLPFYGRTF--KTLASGFLNDVSEGVGFKGPYTREDGFLGYNEICQTLSNQTSGWTREWD  336
             GLPF+GRTF   ++    + D +E   F GPYT  DG++GYNEIC  L+ ++S W + WD
AaCht2  238  GLPFHGRTFIADSIFYTRIGDPAE-TSFSGPYTNIDGYIGYNEICTELNAKSSFWNQAWD  296

DmCht2  337  PQTSQVLAKSERNVFTQEINVVTYDSSRSIANKVLFAMSKRLAGVMVWSVDTDDFLGNCK  396
             P+ S+V+A+ +    T+   V+ YDS+RSIA KV +A+ K L G+M  S+DTDDF GNC
AaCht2  297  PEASEVIARMQDGSKTK---VIVYDSTRSIAAKVRYAVQKNLRGLMASSIDTDDFHGNCN  353

DmCht2  397  LDEDTYEDFQKVTAAPKRSSQNYPLLRTINEATMLAVDEL  436
             +E+T+ DF      P      + LLRTIN A + A DE+
AaCht2  354  AEEETFVDFDNKLRTPPPIQGKFKLLRTINNAIVAAEDEI  393
```

Pediculus humanus corporis (Ph) — *Drosophila melanogaster*

```
DmCht2  33   ARTGPLHDKVVVCYVSTWAVYRPEQGAYAIENFDPNLCTHVVYAFAGLDITQAAIKSLDP  92
               R  P+HDK VVCY+ TW+VYRP +G++ IEN D NLCTH+VYAF GL+ T   ++S+DP
PhCht2  5    TRLKPIHDKHVVCYIGTWSVYRPGKGSFKIENIDGNLCTHIVYAFVGLNATTNTLRSIDP  64

DmCht2  93   WQDLKEEYGKGGYEKMTGLKRSHPHLKVSLAIGGWNEGSANYSTLVANNLLRGRFVKQVS  152
              + DL+E YGKG ++KMT LK  +P+LKV+LA+GGWNEGSANYS +      R +F+  V
PhCht2  65   YYDLEENYGKGSFKKMTQLKLKYPNLKVTLAVGGWNEGSANYSNMALVPENRRKFINSVM  124

DmCht2  153  SFIRKYNFDGLDLDWEYPTQRKGKPADRENFVLLTKELREEFDEHGLLLTSAIGASKKVI  212
              ++ KYNFDG DLDWE+P  R G+P D++NF LL KEL++E  +  L+LT+A+GA+  I
PhCht2  125  EYVTKYNFDGFDLDWEFPAARGGRPEDKQNFALLVKELKQELGKKNLILTAALGAAINTI  184

DmCht2  213  DEAYDVRQISRYLDYLHIMCYDYHGSWDRRVGYNAPLTAPADDPLSVKFSIDYLLKLGAP  272
              + AYDV +IS++LD LH MCYDYHG WD+ VG NAPLT  + D L ++ SI +LL+LGAP
PhCht2  185  NTAYDVPEISKHLDLLHFMCYDYHGPWDKTVGANAPLT--SKDSLDLESSITHLLQLGAP  242

DmCht2  273  PEKLVMGLPFYGRTF--KTLASGFLNDVSEGVGFKGPYTREDGFLGYNEICQTLSNQTSG  330
              P KLV+G+P YG TF  K + +  +    G G +G +T++ GF GYNEIC  L
PhCht2  243  PHKLVVGIPAYGHTFLTKNVTNPKMGTPITGPGPEGVFTKQQGFQGYNEICTELLKDDEK  302

DmCht2  331  WTREWDPQTSQVLAKSERNVFTQEINVVTYDSSRSIANKVLFAMSKRLAGVMVWSVDTDD  390
              W++ WD ++S  A +          +V+ +D  +SI  KV  +  ++ G M+WS+DTDD
PhCht2  303  WSKHWDDESSTPYAVNGN-------HVIAFDDEKSIGEKVNLGLKYKVGGFMIWSIDTDD  355

DmCht2  391  FLGNCKLDEDTYEDFQKVTAAPKRSSQN---YPLLRTINEATMLAVDELAVPEPQPDDSE  447
              F G+C         K     K S+Q+   +PLLR    A  A+ +  +      DD
PhCht2  356  FHGDC----------DKERLKVKESAQDKYTFPLLR----AVHTAIQQYKL----QDDDN  397

DmCht2  448  NEIPHGSIADRKNAGASMV--SLGLGVTAVFMLL  479
              N+IP     D  +   + V  S    +TA F+++
PhCht2  398  NDIPEVGTNDIDDPKENNVVPSFQSSITANFVVI  431
```

DmCht2 (AAF47562.1; see also flybase: http://flybase.org/reports/FBgn0022702.html)

```
  1 mtlrsrlsge apqlwlllll astasslwas vaartgplhd kvvvcyvstw avyrpeqgay
 61 aienfdpnlc thvvyafagl ditqaaiksl dpwqdlkeey gkggyekmtg lkrshphlkv
121 slaiggwneg sanystlvan nllrgrfvkq vssfirkynf dgldldweyp tqrkgkpadr
181 enfvlltkel reefdehgll ltsaigaskk videaydvrq isryldylhi mcydyhgswd
241 rrvgynaplt apaddplsvk fsidyllklg appeklvmgl pfygrtfktl asgflndvse
301 gvgfkgpytr edgflgynei cqtlsnqtsg wtrewdpqts qvlakserny ftqeinvvty
361 dssrsiankv lfamskrlag vmvwsvdtdd flgnckided tyedfqkvta apkrssqnyp
421 llrtineatm lavdelavpe pqpddsenei phgsiadrkn agasmvslgl gvtavfmllh
481 rlaq
```

b

Aedes aegypti (Aa) — *Drosophila melanogaster (Dm)*

```
DmIdgf6  7    AIVSLCLASIQASKVGAPQLPKKHLVCYYDSASFVKEGLGKLVIDELEPALQFCDYLVYG  66
              A+ ++CL  +  +  G  Q   K ++CYYD A+F+ EGLGK+ + ++E AL FC +LVYG
AaIdgf6  7    AVTAICLLGVLLAPTGYSQSTAK-VLCYYDGANFLIEGLGKVSLTDIEAALPFCTHLVYG  65

DmIdgf6  67   YAGIERDSHKAVSLNQQLDLDLGKGLYRTVTRLKRKYPNVKILLSVGGDKDIELDKDAKE  126
              YA ++ DS+KAVS N  LDLD GKG YR VT LKRK+P +K+LL +GG +
AaIdgf6  66   YAAVDPDSNKAVSRNANLDLDTGKGNYRQVTGLKRKFPGLKVLLGIGGYR-------FSA  118

DmIdgf6  127  LPNKYLELLESPTGRTRFVNTVYSLVKTYGFDGLDVAWQFPKNKPKKVHSGIGSLWKGFK  186
                 KYLELLES   R  F+N+VYS+VK Y FDG+D+AWQFP+NKPKKV S  G LW GFK
AaIdgf6  119  ASPKYLELLESGAARITFINSVYSIVKAYDFDGIDLAWQFPQNKPKKVRSSPGKLWHGFK  178

DmIdgf6  187  KVFSGDSIVDEKSEEHKEQFTALLRDVKNAFRPDNLLLSTTVLPNVNSSLFYDIPAVVNY  246
              KVF+GDS++DEK+EH+E+FTALLR++KNAFR D   L  TVL +VN+S+F DIPA+VNY
AaIdgf6  179  KVFTGDSVLDEKADEHREEFTALLREMKNAFRSDGYQLGITVLSHVNASMFMDIPAIVNY  238

DmIdgf6  247  LDFVNLGTFDFFTPQRNPEVADYAAPIYELSERNPEFNVAAQVKYWLRNNCPASKINVGV  306
              LDFVN+  +D  TP+RN + AD+ APIYELSER P  NV   VK WL ++ PASK+ V +
AaIdgf6  239  LDFVNIAAYDMQTPERNKKEADFYAPIYELSERIPGNNVDGLVKVWLNSHAPASKLIVSI  298

DmIdgf6  307  ATYGRPWKLTDDSGDTGVPPVKDVKDEA-PVGGNTQVPGIYSWPEVCALLPNQNNAYLKG  365
               T+GR WK+T+DSG TGVPP+  V D A P G   Q  G YSW E CA LPN +N  LKG
AaIdgf6  299  PTHGRGWKMTEDSGITGVPPL--VADGASPAGTQLQQEGFYSWAETCAKLPNPSNTALKG  356

DmIdgf6  366  ANAPLIKVQDPAKRFGSYAYRAADKKGDNGIWVSFEDPDTAADKAGYVRTENLGGVALFD  425
              A+ PL KV DP KRFGSYA+R  D  G++G+WV++EDPD+A +KA YV+ + LGG+A+ D
AaIdgf6  357  ADGPLRKVGDPTKRFGSYAFRLPDSSGEHGMWVTYEDPDSAGNKAAYVKAKGLGGIAIND  416

DmIdgf6  426  LSYDDFRGLCTNEKYPILRAIKYRL  450
              L++DDFRG C  EKYPILRA KYRL
```

Anopheles gambiae (Ag) — *Drosophila melanogaster (Dm)*

```
DmIdgf6  1    MIIKALAIVSLCLASIQASKVGAPQLPKKHLVCYYDSASFVKEGLGKLVIDELEPALQFC  60
              M  K  A  L L  +  S+    Q P K ++CYYD+A+F+ EGLGK+ + +++ AL FC
AgBRP2   1    MWCKQFAGALLLL--VATSQYVQSQQPSK-VLCYYDAANFLIEGLGKVSLADIDAALPFC  57

DmIdgf6  61   DYLVYGYAGIERDSHKAVSLNQQLDLDLGKGLYRTVTRLKRKYPNVKILLSVGGDKDIEL  120
               +LVYGYAGI+ +++KAVS    LDLD GKG YRTVT+LK KYP++K+LL +GG K
AgBRP2   58   THLVYGYAGIDVETNKAVSRQPNLDLDTGKGNYRTVTQLKSKYPSLKVLLGLGGYK----  113

DmIdgf6  121  DKDAKELPNKYLELLESPTGRTRFVNTVYSLVKTYGFDGLDVAWQFPKNKPKKVHSGIGS  180
                  E   KYL LLES   R  F+N+VYSL+KTYGFDG+D+ WQFP NKPKKV S +G
AgBRP2   114  ---FSEPSIKYLTLLESGAARITFINSVYSLLKTYGFDGVDLEWQFPMNKPKKVRSTLGG  170

DmIdgf6  181  LWKGFKKVFSGDSIVDEKSEEHKEQFTALLRDVKNAFRPDNLLLSTTVLPNVNSSLFYDI  240
              +W GFKKVFSGDS++DEK+EEH+E+FTALLR++KNAFR D   L  TVL +VNSS+F DI
AgBRP2   171  VWHGFKKVFSGDSVLDEKAEEHREEFTALLRELKNAFRSDGYQLGITVLSHVNSSVFMDI  230

DmIdgf6  241  PAVVNYLDFVNLGTFDFFTPQRNPEVADYAAPIYELSERNPEFNVAAQVKYWLRNNCPAS  300
              PA++NYLDFVN+  +D  TP RN + AD+AAP+YELS+R P  NV  QV+ WL NN PAS
AgBRP2   231  PAIINYLDFVNIAAYDQQTPTRNKKEADHAAPLYELSDRVPGNNVDGQVRLWLTNNAPAS  290

DmIdgf6  301  KINVGVATYGRPWKLTDDSGDTGVPPVKDVKDEAPVGGNTQVPGIYSWPEVCALLPNQNN  360
              K+ V + T+GR WK+  DSG TGVPP+        P G  TQ  G YSW EVCA+LPN +N
AgBRP2   291  KLIVSIPTFGRGWKMNGDSGITGVPPLPADGPSNP-GPQTQTEGFYSWAEVCAMLPNPSN  349

DmIdgf6  361  AYLKGANAPLIKVQDPAKRFGSYAYRAADKKGDNGIWVSFEDPDTAADKAGYVRTENLGG  420
                LKGA+APL KV DP KRFGSYA+R  D  G++G+WVS+EDPDTA +KAGYV+ +NLGG
AgBRP2   350  TALKGADAPLRKVGDPTKRFGSYAFRLPDSNGEHGVWVSYEDPDTAGNKAGYVKAKNLGG  409

DmIdgf6  421  VALFDLSYDDFRGLCTNEKYPILRAIKYRL  450
              +A+ DLSYDDFRG C  EK+PILRA KYRL
AgBRP2   410  IAINDLSYDDFRGSCAGEKFPILRAAKYRL  439
```

DmIdgf6, (NP_477081.1; see also flybase: http://flybase.org/reports/FBgn0013763.html

```
  1 miikalaivs lclasiqask vgapqlpkkh lvcyydsasf vkeglgklvi delepalqfc
 61 dylvygyagi erdshkavsl nqqldldlgk glyrtvtrlk rkypnvkill svggdkdiel
121 dkdakelpnk ylellesptg rtrfvntvys lvktygfdgl dvawqfpknk pkkvhsgigs
181 lwkgfkkvfs gdsivdekse ehkeqftall rdvknafrpd nlllsttvlp nvnsslfydi
241 pavvnyldfv nlgtfdfftp qrnpevadya apiyelsern pefnvaaqvk ywlrnncpas
301 kinvgvatyg rpwkltddsg dtgvppvkdv kdeapvggnt qvpgiyswpe vcallpnqnn
361 aylkganapl ikvqdpakrf gsyayraadk kgdngiwvsf edpdtaadka gyvrtenlgg
421 valfdlsydd frglctneky pilraikyrl tn
```

Cht2 and Idgf6 amino acid sequence analysis shows non-conserved regions.

(a) The Cht2 C-terminal (a) and the Idgf6 N-terminal (b) amino acid sequences are not conserved in insects. Alignments compare amino acid sequences of *Drosophila*, mosquitos and other harmful insects. The amino acids marked in red are less conserved. Non conserved sequences are potential candidate target regions for a species specific insecticide. *Drosophila melanogaster* Cht2 (AAF47562.1); *Pediculus humanis corporis* (body louse, ectoparasite and transmitter of epidemic typhus; XP_002423089.1); *Aedes aegypti* (Cht2, XP_001657537.1; Idgf6, XP_001660748.1) and *Anopheles gambiae* (bacteria responsive protein2 (BRP2; AAS80138.1)). Blast search and alignments by NCBI database.

Supplementary Table 1

Gene	Primer	sequence	efficiency	splice var., #no.
rp49	for	GCTAAGCTGTCGCACAAATG	100%	
rp49	rev	GTTCGATCCGTAACCGATGT		
Cht2	for	ACGAGAAAATGACGGGTCTG	98%	detects all, #6
Cht2	rev	TGAAACTGGACACCTGCTTG		
Cht4	for	ATGACAATCCCGAAAGCATC	105%	
Cht4	rev	ACAGACCACGGAAATCATCC		
Cht5	for	CAACAACCACCACAACAACC	94%	detects all, #2
Cht5	rev	ATCTCGTTGGGATCGAACTG		
Cht6	for	ATGCCCAGTTTGTCCAAGAG	99%	detects all, #14
Cht6	rev	AACTTGGGCACATCGTAACC		
Cht7	for	TTACGAGGCCATCGACTTTC	91%	detects all, #2
Cht7	rev	CCACTCGCAATTCCTTTAGC		
Cht8	for	TCTGGGCAAGTTCGGTATG	70%	detects all, #2
Cht8	rev	GCGTCCACTATTCTCCTCAAG		
Cht9	for	TCGTGCCAACTTTGTGACTC	90%	detects all, #2
Cht9	rev	CGATTTCGTAGGATGCACTG		
Cht11	for	CTGCAAGGCGAACTATGTGA	93%	
Cht11	rev	TGGGTTAGCGGAAATACAGG		
Cht12	for	TCGGACTTTATCCACCTGATG	75%	detects all, #2<br>red= intron
Cht12	rev	CTCCTCCATTTCGTTTCCAG		

The table 1 summarizes used primer sequences and their efficiencies for Chts. The Cht12 forward primer recognizes exon (black) and intron (red) sequences. The table further shows the gene splicing variants and if they are detected by the primer pairs.

Supplementary Table 2

Gene	Primer	sequence	efficiency	splice var., #no.
idgf1	for	CAGCTCCGATTTTCTTCCAG	97%	detects all, #2
idgf1	rev	TCCATGCTCTGCCATAACTG		
idgf3	for	TGGAACACTGGTTGCTTCAG	97%	detects all, #8
idgf3	rev	CCGAATCCTTGCTCATCTTC		
idgf4	for	GAAATTGGACCTGGATCTGG	99%	red, detects all, #6
idgf4	rev	CAGCAGGGTCAGATACTTGTTG		
idgf5	for	ACCAGACATCCGAAATCAGTG	94%	detects all, #2
idgf5	rev	TCGGGTCATATTCCAAGCTC		
idgf6	for	AGCGCAAGTATCCCAATGTC	88%	detects all, #4
idgf6	rev	CGGTATTCACGAAACGAGTG		

The table 2 summarizes used primer sequences and their efficiencies for idgfs. The idgf4 forward primer sequences marked in red detects all predicted splicing variants. The black labeled part detects only 5 variants. The table further indicates the gene splicing variants and if they are recognized by the primer pairs.

Supplementary Table 3

Gene	web link for FlyBase gene card
Cht2	http://flybase.org/reports/FBgn0022702.html
Cht4	http://flybase.org/reports/FBgn0022700.html
Cht5	http://flybase.org/reports/FBgn0038180.html
Cht6	http://flybase.org/reports/FBgn0263132.html
Cht7	http://flybase.org/reports/FBgn0035398.html
Cht8	http://flybase.org/reports/FBgn0034580.html
Cht9	http://flybase.org/reports/FBgn0034582.html
Cht11	http://flybase.org/reports/FBgn0029913.html
Cht12	http://flybase.org/reports/FBgn0050293.html
idgf1	http://flybase.org/reports/FBgn0020416.html
idgf3	http://flybase.org/reports/FBgn0020414.html
idgf4	http://flybase.org/reports/FBgn0026415.html
idgf5	http://flybase.org/reports/FBgn0064237.html
idgf6	http://flybase.org/reports/FBgn0013763.html

Table 3 provides the direct link to FlyBase

Supplementary References

1. Zhu, Q. Arakane, Y. Beeman, R. W. Kramer, K. J. & Muthukrishnan, S. Functional specialization among insect chitinase family genes revealed by RNA interference, *Proceedings of the National Academy of Sciences* **105,** 6650–6655 (2008).

2. Ohlen, T. von, Luce-Fedrow, A. Ortega, M. T. Ganta, R. R. & Chapes, S. K. Identification of Critical Host Mitochondrion-Associated Genes during Ehrlichia chaffeensis Infections, *Infection and Immunity* **80,** 3576–3586 (2012).

3. Wingen, C. Stümpges, B. & Hoch, M and Behr, M. Expression and localization of clathrin heavy chain in Drosophila melanogaster, *Gene Expr. Patterns* **9,** 549–554 (2009).
